# Randomized phase 3 open label study of quality of life of patients on Pemetrexed versus Erlotinib as maintenance therapy for advanced non squamous non EGFR mutated non small cell lung cancer

**DOI:** 10.18632/oncotarget.27214

**Published:** 2019-10-29

**Authors:** Vijay Patil, Amit Joshi, Vanita Noronha, Vivek Agarwala, Anuradha Chougule, Sadhana Kanan, Atanu Bhattacharjee, Arun Chandrasekharan, Nikhil Pande, Vijai Simha, Supriya Goud, Sucheta More, Rajiv Kumar, Abhishek Mahajan, Amit Janu, Nilendu Purandare, Kumar Prabhash

**Affiliations:** ^1^ Department of Medical Oncology, Tata Memorial Hospital, Mumbai, India; ^2^ Department of Biostatistics, Advanced Center for Treatment, Research, and Education in Cancer, Mumbai, India; ^3^ Section of Biostatistics, Centre for Cancer Epidemiology, Tata Memorial Hospital, Mumbai, India; ^4^ Department of Pathology, Tata Memorial Hospital, Mumbai, India; ^5^ Department of Radiology, Tata Memorial Hospital, Mumbai, India; ^6^ Department of Nuclear Medicine, Tata Memorial Hospital, Mumbai, India; ^7^ Homi Bhabha National Institute, Mumbai, India

**Keywords:** maintenance, pemetrexed, erlotinib, NSCLC

## Abstract

**Background:** We planned to compare pemetrexed maintenance with erlotinib maintenance in non squamous non Epidermal Growth Factor Receptor (EGFR) mutated non small cell lung cancer (NSCLC). The null hypothesis for this study was that there would be no difference in quality of life (QOL) between pemetrexed and erlotinib maintenance.

**Results:** The QL2 scores at 3 months were 63.35 (SD 24.99) in pemetrexed arm and 63.01(SD 23.04) in erlotinib arm (p-0.793). Except in 1 domain, the scores were statistically similar between the 2 arms. In the domain of diarrhea, the score was higher as expected in the erlotinib arm (p-0.048). The median progression free survival was 4.5 months (95%CI 4.1–4.9 months) in pemetrexed arm versus 4.5 months (95%CI 3.8–5.2 months) in erlotinib arm (p-0.94). The median overall survival was 16.6 months (15.2–17.9 months) in pemetrexed arm versus 18.3 months (95% CI 13.75–22.91 months) in erlotinib arm (p-0.49).

**Methods:** The study was an open label, single centre, parallel, phase 3 randomized study with 1:1 randomization between maintenance pemetrexed arm and erlotinib arm. Adult patients (age > or = 18 years), with non squamous EGFR mutation, treated with first line palliative therapy, with non progressive disease post 4–6 cycles of pemetrexed-carboplatin were randomized. Primary outcome was change in the score of QOL (Global health status {QL2}) at 3 months. We estimated that with 200 patients, the study had 80% power to detect a significant difference between the two groups in the change in the global health status score at 3 months with an alpha error of 5%, with an effect size of 0.3 SD.

**Conclusions:** Maintenance pemetrexed post pemetrexed-platinum chemotherapy fails to improve QOL or time to event outcomes over maintenance erlotinib in EGFR mutation negative NSCLC.

## INTRODUCTION

Treatment of non small cell lung cancer (NSCLC) has evolved over the last one and a half decades. Identification of driver mutations and treatment of these mutated cancers with appropriate tyrosine kinase inhibitors has led to encouraging improvements in outcomes [1–3]. Progress has been made in the management of non driver mutated non squamous NSCLC as well. Pemetrexed-cisplatin regimen is now considered as the favoured regimen for first line treatment in multiple guidelines [4, 5]. This recommendation is based on a phase 3 study reported by Scagliotti et al where there was an overall survival (OS) advantage over gemcitabine cisplatin for non squamous histologies [6]. In addition, this advantage came with lower frequency of adverse events. There have been advances with regard to maintenance treatment as well. Multiple studies have suggested that maintenance with pemetrexed either as continuation after pemetrexed-platinum therapy or as switch therapy after platinum doublet was feasible and improved survival [7–9]. Maintenance therapy with pemetrexed led to an improvement in median OS by 5.2 months (HR 0.70, *p* ≤ 0.0001) in the study reported by Ciuleanu *et al*. [10] Such benefits with chemotherapy were unheard of in NSCLC.

Maintenance with pemetrexed, though beneficial, has issues. None of these initial studies have stratified the patients based on their driver mutation status. Further, the intravenous delivery of pemetrexed maintenance meant recurring and continuous hospital visits for the patient. Such frequent visits can be physically and financially distressing and can also lead to non-compliance. Also, there is inconclusive data regarding the cost effectiveness of pemetrexed maintenance [11, 12]. These issues with intravenous maintenance therapy can be overcome by having an oral substitute.

Erlotinib is a reversible tyrosine kinase inhibitor which is approved for treatment of Epidermal Growth Factor Receptor (EGFR) mutation positive NSCLC [13]. However, multiple studies have also shown erlotinib to be active in EGFR activating mutation negative (EGFR neg) patients. In the SATURN study, switch maintenance with erlotinib was associated with a survival benefit, even in EGFR neg patients [14]. With this background, we planned to compare pemetrexed maintenance with erlotinib maintenance in non squamous EGFR neg NSCLC. The null hypothesis for this study was that there would be no difference in quality of life (QOL) between pemetrexed maintenance and erlotinib maintenance.

### Highlights

1) We compared erlotinib and pemetrexed as maintenance therapy; 2) There was no difference in QOL between the 2 arms; 3) The PFS and OS were similar between the 2 arms.

## RESULTS

### Baseline characteristics

**Figure 1:**
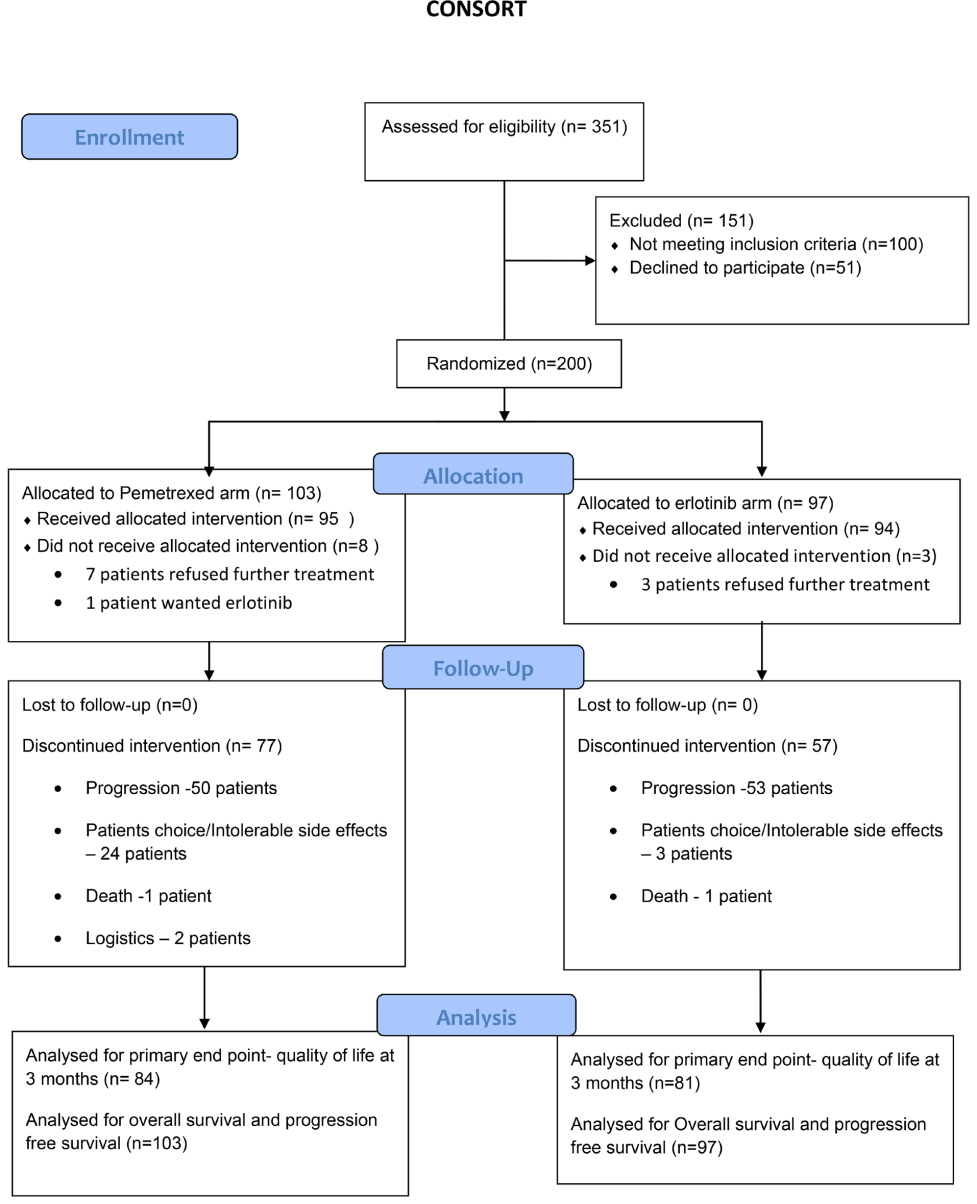
Consort diagram.

200 patients were recruited in the above mentioned study period. The consort diagram is shown in Figure 1. The median age of the patient on pemetrexed arm and erlotinib arm were 55 years (28–76 years) & 56 years (28–79 years), respectively. All patients had received induction therapy with pemetrexed carboplatin. The stage of these patients at the start of induction pemetrexed -carboplatin was stage IIIB in 11 patients (5.5%) and stage IV in 189 patients (94.5%), respectively. The response rate post induction therapy was 41.8% and 29.9%, respectively, in pemetrexed arm and erlotinib arm, respectively. Table 1 depicts the baseline characteristics in both arms.

**Table 1:** Table depicting the baseline characteristics in both arms

Arm variables ↧	Pemetrexed arm (*n* = 103)	Erlotinib arm (*n* = 97)	Overall
Median age	55 years (28–76)	56 years (28–79)	55 years (28–79)
Gender			
Male	71 (68.9%)	61 (62.9%)	132 (66.7%)
Female	32 (31.1%)	36 (37.1%)	68 (39.0%)
ECOG PS			
0–1	98 (95.2%)	92 (94.8%)	190 (95.0%)
2	01 (1.0%)	—	1 (0.5%)
Missing	04 (3.8%)	5 (5.2%)	9 (4.5%)
Smoking history			
Never smoker	53 (51.5%)	54 (55.7%)	107 (53.5%)
Previous smoker	50 (48.5%)	43 (44.3%)	93 (46.5%)
Stage			
IIIB	6 (5.8%)	5 (5.2%)	11 (5.5%)
IV	97 (94.2%)	92 (94.8%)	189 (94.5%)
Sites of metastasis			
Brain	15 (14.6%)	12 (12.4%)	27 (13.5%)
Bone	40 (38.8%)	39 (40.2%)	79 (39.5%)
Liver	21 (20.4%)	15 (15.5%)	36 (18.0%)
Response to induction therapy			
Complete response	1 (1%)	—	1 (0.5%)
Partial response	42 (40.8%)	29(29.9%)	71 (35.5%)
Stable disease	60 (58.3%)	68 (70.1%)	128 (64%)

### Compliance

Out of 103 patients randomized on pemetrexed arm, 95 patients (92.2%) received at least one cycle of chemotherapy. The median number of cycles of pemetrexed received were 5 (1–33). Pemetrexed was stopped in 77 patients (74.8%). The reasons for stopping pemetrexed were progression in 50 patients, patient’s choice or intolerable side effects in 24 patients, for logistic reasons in 2 patients and death before progression in 1 patient.

Out of 97 patients randomized on erlotinib arm, 94 patients (96.9%) took at least one week of erlotinib. Erlotinib was stopped in 83 patients (85.5%). The reason for stopping erlotinib were progression in 65 patients, patient’s choice or intolerable side effects in 17 patients and death before progression in 1 patient.

### QOL

One hundred and sixty five patients were eligible for primary endpoint analysis. The baseline QOL scores are shown in Table 2, they were comparable between the 2 arms. The QOL scores in each domain at 3 months are shown in Table 3. The data of QOL scores at 3 months were available for 165 patients. The global health status QOL scores at 3 months were 63.35 (SD 24.99) in pemetrexed arm and 63.01(SD 23.04) in erlotinib arm (p-0.793). Except in 1 domain, the scores were statistically similar between the 2 arms. In the domain of diarrhoea, the score was higher in the erlotinib arm (p-0.048). There was no difference in time to deterioration between any QOL domains between the 2 arms.

**Table 2:** Domain wise QOL score value comparison between Pemetrexed and Erlotinib

Domain	Means (SD) in Pemetrexed	Means (SD) in Erlotinib	*P*-value
Global health status	62.90 (24.74)	63.65 (22.76)	0.692
Physical functioning	76.57 (17.83)	76.714 (16.76)	0.919
Role functioning	84.13 (23.07)	87.29 (21.02)	0.074
Emotional functioning	71.11 (22.43)	73.64 (23.49)	0.167
Cognitive functioning	83.75 (18.35)	84.63 (20.52)	0.569
Social functioning	82.49 (24.91)	85.76 (23.46)	0.092
Fatigue	33.58 (23.00)	32.70 (22.66)	0.629
Nausea and vomiting	15.50 (20.11)	14.13 (17.68)	0.368
Pain	20.37 (21.54)	23.40 (23.44)	0.089
Dyspnoea	21.63 (25.33)	20.57 (23.91)	0.59
Insomnia	18.45 (26.00)	21.04 (27.95)	0.22
Appetite loss	23.22 (26.78)	22.58 (27.81)	0.767
Constipation	14.98 (25.39)	12.29 (22.98)	0.167
Diarrhoea	8.61 (18.57)	12.88 (23.26)	0.010
Financial difficulties	43.26 (34.59)	39.83 (33.05)	0.206
Dyspnoea	23.34 (18.44)	22.10 (19.78)	0.41
Coughing	35.29 (30.42)	35.22 (29.16)	0.97
Haemoptysis	3.27 (11.94)	3.07 (11.52)	0.82
Sore mouth	10.48 (19.91)	13.59 (23.19)	0.06
Dysphagia	9.17 (18.30)	6.97 (18.07)	0.13
Peripheral neuropathy	16.57 (23.93)	18.91 (25.55)	0.23
Alopecia	18.91 (27.59)	21.04 (29.06)	0.34
Pain in chest	13.57 (21.95)	15.48 (23.70)	0.29
Pain in arm or shoulder	12.73 (21.52)	14.06 (23.76)	0.45
Pain in other parts	17.04 (28.02)	17.61 (26.84)	0.79

**Table 3:** Domain wise QOL score value comparison between Pemetrexed and Erlotinib at 3 months

Domain	Means (SD) in Pemetrexed	Means (SD) in Erlotinib	*P*-value
Global health status	63.35 (24.99)	63.01(23.04)	0.793
Physical functioning	76.61(16.96)	76.96(16.82)	0.848
Role functioning	85.28(21.86)	86.06(22.48)	0.746
Emotional functioning	73.11(24.06)	71.39(22.45)	0.501
Cognitive functioning	84.66(20.28)	86.45(16.76)	0.379
Social functioning	83.44(27.31)	85.67(21.94)	0.409
Fatigue	34.08(24.10)	32.75(22.09)	0.598
Nausea and vomiting	12.58(17.38)	14.81(17.47)	0.241
Pain	23.42(25.63)	23.00(22.37)	0.875
Dyspnoea	24.74 (27.86)	19.30(29.93)	0.055
Insomnia	20.86(26.98)	21.83(31.16)	0.760
Appetite loss	22.70 (27.14)	23.78(29.25)	0.727
Constipation	16.97(28.53)	12.87(25.88)	0.169
Diarrhoea	7.15 (18.04)	11.31(20.18)	0.048
Financial difficulties	43.35(33.97)	40.74 (35.38)	0.492
Dyspnoea	23.44(20.62)	22.98(19.30)	0.830
Coughing	39.46(33.58)	33.71(27.67)	0.085
Haemoptysis	2.45(9.48)	4.19(14.12)	0.188
Sore mouth	10.83(20.91)	12.38(22.72)	0.517
Dysphagia	5.72(15.97)	9.14(20.34)	0.088
Peripheral neuropathy	14.31(21.90)	16.95(25.73)	0.312
Alopecia	20.85(28.70)	18.28(26.42)	0.391
Pain in chest	15.13(26.75)	15.42(21.66)	0.910
Pain in arm or shoulder	9.20(17.48)	16(25.97)	0.005
Pain in other parts	15.54(27.03)	15.42(26.44)	0.968

The data for adverse events was available for 95 patients in the pemetrexed arm and 89 patients in the erlotinib arm. The rate of any grade adverse event between the 2 arms was seen in 87.5% (*n* = 84) and 97.8% (*n* = 87) in pemetrexed arm and erlotinib arm, respectively (p-0.011). The rate of grade 3 or above adverse events between the 2 arms were 8.3% (*n* = 8) and 20.2% (*n* = 18), respectively. There was a difference in the types of adverse events between the 2 arms. The rate of hematological adverse events was higher in the pemetrexed arm, while that of dermatological adverse events was higher in erlotinib arm. The adverse events between the 2 arms are shown in Table 4.

**Table 4:** Grade 3 or above adverse events between the 2 arms

Grade 3-5 adverse events	Pemetrexed arm (*n* = 95)	Erlotinib arm (*n* = 89)
Anemia	4 (4.2%)	2 (2.3%)
Neutropenia	3 (3.2%)	—
Thrombocytopenia	1 (1.1%)	2 (2.3%)
SGPT rise	—	1 (1.1%)
Diarrhea	—	4 (4.9%)
Skin rash	1 (1.1%)	20 (22.5%)
Mucositis	—	2 (2.3%)
Pruritus	—	2 (2.3%)

### Time to event outcomes

**Figure 2:**
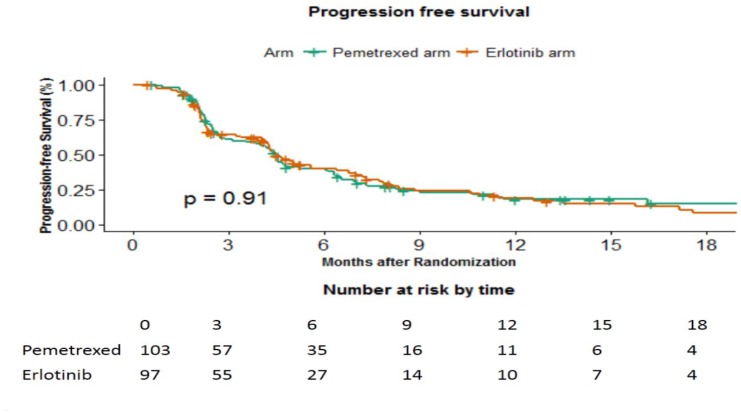
Progression free survival in months between the 2 arms.

The median follow up was 25.36 months. At time of censoring the data, progression was noted in 75 patients in pemetrexed and 70 patients in the erlotinib arm, respectively. The median PFS was 4.5 months (95%CI 4.1–4.9 months) in pemetrexed arm versus 4.5 months (95%CI 3.8–5.2 months) in the erlotinib arm (p-0.94) (Figure 2). The corresponding hazard ratio was 0.982 (95%CI 0.709–1.361).

**Figure 3:**
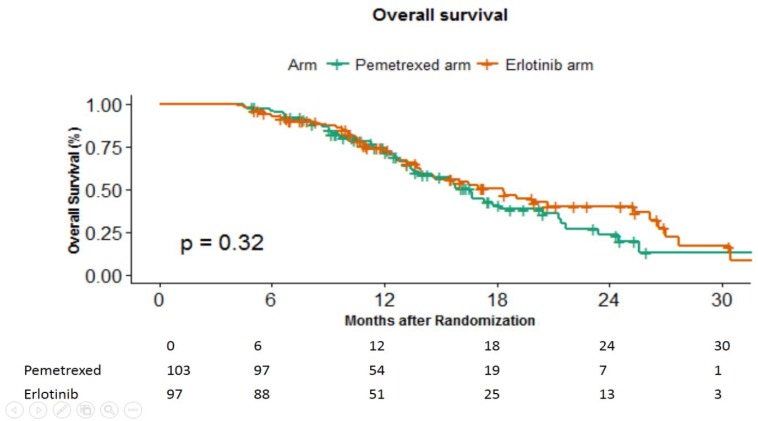
Overall survival in months between the 2 arms.

There were 53 deaths in pemetrexed and 48 deaths in erlotinib arm, respectively. The median OS was 16.6 months (15.2–17.9 months) in pemetrexed arm versus 18.3 months (95% CI 13.75–22.91 months) in erlotinib arm (p-0.49) (Figure 3). The corresponding hazard ratio was 1.222 (95% CI 0.821–1.818).

## DISCUSSION

The current study upheld the null hypothesis. It clarified that QOL and time to event outcomes (PFS and OS) with continuation of maintenance pemetrexed after pemetrexed carboplatin doublet are similar to those obtained with administration of erlotinib as switch maintenance. These results are a surprise and they challenge the current favored regimen of pemetrexed maintenance after pemetrexed -carboplatin. It renews interest once again in the use of erlotinib in EGFR activating mutation negative NSCLC.

The saga of erlotinib in EGFR activating mutation negative cancer started with the publication of BR.21 results. In this study, patients who had failed one or more lines of chemotherapy had received either erlotinib or placebo. Erlotinib, in comparison with placebo, led to an improvement in hazard of progression (hazard ratio, 0.61, adjusted for stratification categories; *P* < 0.001) and decreased the hazard of death too (hazard ratio, 0.70; *P* < 0.001) [15]. Erlotinib was compared against second line chemotherapy agents (standard docetaxel or pemetrexed regimens, at the treating investigators’ discretion) in the TITAN study. The median PFS and OS was similar between the 2 arms [17], thus paving way for use of erlotinib in this setting. Both BR.21 and TITAN study were conducted in predominantly EGFR activating mutation negative population. The median PFS in BR.21 and the TITAN study are similar to that seen in our study. In addition, the impact of erlotinib in the maintenance setting against placebo was shown in the SATURN study. Maintenance erlotinib in the SATURN study, irrespective of EGFR activating mutation status led to an improvement in progression free survival [14]. Our study took this a step forward, and demonstrated that even in maintenance setting, erlotinib has activity similar to that of maintenance pemetrexed. Taking all these studies and ours together, it seems that erlotinib has activity in EGFR mutation negative patients and has activity comparable to chemotherapy agents.

The results of our study need to be discussed in consideration with the recent Food and Drug Administration (FDA-United states) decision of removing erlotinib label in EGFR activating mutation negative NSCLC. This decision was based on the recently reported IUNO study. In this study, non EGFR mutated patients post 4 cycles of platinum doublet were randomized to either early erlotinib (maintenance) or placebo. Patients in placebo arm were treated with erlotinib on progression, thus effectively making it a comparison of early or maintenance erlotinib versus delayed or second line erlotinib. In this study, there was no impact of early erlotinib on OS, PFS or even disease control rate [18]. Thus the authors concluded that erlotinib should not be used in non EGFR mutated patients. The results of our study and the SATURN study, however, suggest the contrary. The probable reasons for these discrepancies are detailed below. Firstly, the primary endpoint of the IUNO study was OS which is an endpoint influenced by multiple factors, the important one being second line or subsequent therapy. Second line therapy was received by only 50% of patients from the maintenance erlotinib arm while 78% received it from the maintenance placebo arm. This was in spite of similar rate of progression between both arms. Secondly, the median PFS in the IUNO study was similar in placebo and erlotinib arm. This is an odd finding, considering that 2 previous studies, BR.21 and SATURN, showed an improvement with erlotinib over placebo [14, 15]. This raises the possibility that such a finding could be a result of either statistical chance or population pharmacogenomics. Whenever trials are performed, the false negative is set at 10–20%. In the IUNO study, it was 20%. Thus there exists a 20% probability that the results found could be falsely negative. Population pharmacogenomics can further play a role. IUNO was a predominantly east european and east asian study. The pharmacogenomics of these populations are different from Indians and Western population [19]. Pharmacogenetic variation is known in erlotinib and can influence both response and toxicity [20–22].

The current guideline is to consider immunotherapy in first line setting in NSCLC and hence the importance of our results can be questioned. However, PDL1 expression is required for administration of immunotherapy. Pembrolizumab alone can be administered if PDL1 expression is > 50%. For PDL1 expression of 1–49% a combination of chemotherapy with immunotherapy is required. Hence, chemotherapy without immunotherapy will still be used in patients without driver mutation and PDL1 expression below 1% [4]. In multiple studies the PDL1 expression <1% is seen roughly in 34–47% of patients [23–25] and hence there will be a substantial proportion of patients who are still candidates for chemotherapy. Accessibility of immunotherapy agents in low and middle income countries is poor [26], and chemotherapy will be the preferred option in these settings.

Adverse event rate seen with maintenance pemetrexed and erlotinib were in accordance with their known side effects. No new safety signals were identified. Pemetrexed was associated with higher rates of myelosuppression, while erlotinib was associated with higher diarrhea rate.

Our study is not without its own limitations. The primary endpoint was quality of life and not PFS or OS. This primary endpoint was selected, as maintenance of QOL in palliative setting was considered as an important aspect of treatment [27]. Improvement in quality of life reflected by symptom control is considered a crucial aspect of maintenance treatment by 90% of patients [28]. It rarely happens that the results of QOL and time to event outcomes are in different directions. The study randomization was not stratified, hence a higher number of patients in the pemetrexed arm had a response to induction chemotherapy. However despite this imbalance, the pemetrexed arm failed to improve QOL, PFS or OS over the erlotinib arm. There was no placebo arm in the study. At the time of conceptualization of the study, maintenance post 4-6 cycles of pemetrexed-platinum was the standard treatment. Both pemetrexed and erlotinib were approved at that time. Hence the lung medical oncology group felt it was unethical to deny patient of maintenance and use a placebo.

## MATERIALS AND METHODS

### Study conduct

The study was approved by the institutional ethics committee and was conducted in accordance with the ethical norms laid down by declaration of Helsinki, good clinical practice guidelines and local institutional ethics guidelines. The study was registered with CTRI India in August 2014 (CTRI/2014/08/004847). The study was conducted in the department of Medical Oncology at Tata Memorial Hospital, Mumbai, India between 7th November 2014 to 3rd March 2017. All patients recruited in this study provided written informed consent prior to participation in the study. The study was funded by an intramural grant from Tata Memorial centre- Research Administrative Council (TRAC).

### Trial design

The study was an open label, single centre, parallel, phase 3 randomized study with 1:1 randomization between maintenance pemetrexed arm and erlotinib arm. There was no major amendment to the study protocol (Supplementary Appendix 1, see Supplementary Materials) or consent post start of the study.

### Participants

The detailed inclusion and exclusion criteria are provided in the study protocol. In brief, adult patients (age > or = 18 years) with histologically proven NSCLC (Non squamous), without activating EGFR mutation, with stage IIIB (not suitable for curative intent therapy) or IV, treated with first line palliative therapy, with non progressive disease post 4–6 cycles of pemetrexed-carboplatin, with normal organ functions (absolute neutrophil count >1500/lL, hemoglobin > 8 g/dL, and platelet count >100,000/lL, serum creatinine <2 mg/dL, total bilirubin <1.5 times the institutional upper limit of normal [ULN], aspartate aminotransferase and alanine aminotransferase levels <2 times the institutional ULN), with Eastern Cooperative Oncology Group (ECOG) performance status (PS) of 0–2 and life expectancy of greater than 3 months were included in this study. Patients who had previous exposure to tyrosine kinase inhibitors, who had uncontrolled comorbidities, who had previous treatment for any other cancer or those with uncontrolled infections were excluded.

### Randomisation

Patients post consenting and workup were 1:1 randomized to the 2 intervention arms. The sequence generation was done by SK, who served as an independent statistician. The randomization had no stratification factor. The request for randomization was done by the trial coordinator online via email and the randomization was performed and conveyed via email by SK.

### Interventions

The 2 intervention arms in the study were maintenance pemetrexed and erlotinib.

Patients randomized to pemetrexed arm received maintenance intravenous pemetrexed. Pemetrexed was administered intravenously every 3 weekly in a dose of 500 mg/m2 over 10–15 minutes. Patients received dexamethasone 8 mg intravenous single dose as antiemetic before pemetrexed. They also received vitamin B12 injection 1000 microgram every 9 weeks and tablet folic acid 5 mg daily while they were on pemetrexed. Patients in the erlotinib arm received tablet erlotinib 150 mg per oral (PO) once daily. Both these interventions were continued till progression of disease or intolerable side effects.

### Endpoints

Primary endpoint of the study was to compare the QOL in both arms at 3 months post randomisation. The secondary endpoints were to compare the progression free survival (PFS), OS and toxicity between the 2 arms. The PFS was defined as time in months from the date of randomization to date of progression or death whichever occurred earlier. The OS was defined as time in months from date of start of pemetrexed-platinum chemotherapy to date of death.

### Study methodology

Patients in both arms, were followed up till death. The dose modifications in both arms were in line with published literature [6, 15]. Patients in both arms underwent response assessment according to RECIST criteria version 1.1 at 2 monthly intervals. Toxicity was documented in accordance with common terminology criteria for adverse events (CTCAE) version 4.03. European Organisation for Research and Treatment of Cancer (EORTC) QOL scale was used for assessment at baseline and at 3 months using the EORTC-QLQ-C30 and LC13 questionnaire for lung cancer.

### Sample size estimation

Primary outcome was change in the score of QOL (Global health status {QL2}) at 3 months. We estimated that with 200 patients, the study had 80% power to detect a significant difference in the change in the global health status score at 3 months with an alpha error of 5%, with an effect size of 0.3 standard deviation (SD).

### Statistical method

#### Quality of life analysis

The quality of life data was analysed using R software using the “QoLR” package. The missing data was examined using the method suggested by Little *et al*. [16] It suggested that the data was not missing completely at random. To overcome the chance of bias from the missing data, it was imputed using multivariate imputation by chained equations algorithm using the “mice” package in R. Imputation was not performed for completely missing data for example if QOL was not filled for any reason at 3 months, it was not imputed. The estimation of the scores of EORTC HRQOL questionnaires QLQ-C30 and EORTC QLQ-LC13 questionnaires at baseline and each visit were in accordance with the method defined in the EORTC scoring manual. The mean scores with the standard deviation were calculated. The scores at 3 months were compared using 2 sided student t test for independent samples. Per protocol analysis was performed for the primary endpoint only those patients who had filled the baseline and subsequent 3 month QOL proforma were included.

The QOL data was also collected for visits in addition to the baseline visit and the 3 month visit. The mean scores between all visits between the 2 arms were calculated. A time to deterioration analysis was planned to estimate the time until definitive deterioration. The time until definitive deterioration (TUDD) in QOl for a specific domain was defined as time interval in months calculated between time to randomization to time to deterioration in that specific QOL domain by 10 or more units or death or progression whichever occurred earlier. The TUDD was compared between the 2 arms for each domain of QOL using log rank test. The A p value of 0.05 was taken as significant and no multiplicity correction was done.

#### Time to event analysis

Median follow up was calculated using the reverse Kaplan Meier method. Intention to treat analysis was performed for PFS and OS. The PFS and OS were estimated using the Kaplan Meier method and the 2 arms were compared using the log rank test. The cox regression analysis was used for calculation of the hazard ratio.

## CONCLUSIONS

In conclusion, to our knowledge, the current study is the first study to show that maintenance pemetrexed post pemetrexed-carboplatin chemotherapy fails to improve QOL or time to event outcomes (OS and PFS) over maintenance erlotinib in EGFR mutation negative NSCLC.

## SUPPLEMENTARY MATERIALS


