# Supplementary material for: Randomized phase 3 open label study of quality of life of patients on Pemetrexed versus Erlotinib as maintenance therapy for advanced non squamous non EGFR mutated non small cell lung cancer
Source: Oncotarget. 2019 Oct 29;10(59):6297–307. doi: 10.18632/oncotarget.27214 (PMC6824869; doi:10.18632/oncotarget.27214)
Supplement: Supplementary file 1 [file oncotarget-10-6297-s001.pdf]

Randomized phase 3 open label study of quality of life of patients on Pemetrexed versus Erlotinib as maintenance therapy for advanced non squamous non EGFR mutated non small cell lung cancer

SUPPLEMENTARY MATERIALS

PROTOCOL

Title: Comparative study of QOL of patients on Pemetrexed versus Erlotinib in maintenance therapy for advanced NSCLC (other than squamous cell carcinoma)

PI: Professor **Prabhash K, Department of Medical Oncology, Tata Memorial Hospital, Mumbai, India**

TABLE OF CONTENTS

Introduction

Aims and Objective

Outcomes

Materials and Methods

Study design

Study population

Inclusion criteria

Exclusion criteria

Treatment Drug

Concomitant Drugs

Dose Modification& Treatment Interruption

Discontinuation of Treatment

Tumour Assessment

Safety and Toxicity Assessment

Statistical Analysis

Feasibility of the Study

Ethical Considerations

Treatment Scheme

References

Appendix 1 Patient Information Sheet &Consent Form

Appendix 2 CTCAE Version 4.03

Appendix 3 EORTC QLQ C-30 and LC 13 Questionnaires

INTRODUCTION

Background

Lung cancer is the leading cause of death from cancer worldwide, with an estimated 1.8 million new cases in 2012 accounting for 13% of all cancer diagnosis [1]. In India, approximately 63,000 new lung cancer cases are reported each year [2]. More than 87% of the cases of lung cancer arenon-small-cell lung cancer [3]. Forty percent of patients with newly diagnosed non-small cell lung cancer (NSCLC) have either stage IIIB disease with malignant effusion or stage IV disease [4]. Treatment goals are to prolong survival, control disease-related symptoms and to improve quality of life (QOL), all of equal importance. Treatment options include cytotoxic chemotherapy and targeted agents. Radiation therapy and surgery are generally used in selective cases for symptom palliation. Despite significant advances in the treatment of advanced non-small cell lung cancer (NSCLC), overall prognosis remains poor. Maintenance therapy in advanced NSCLC has gained much recognition as a possible treatment strategy.

Standard treatment options for advanced NSCLC

Platinum-based chemotherapy represents the standard of care for patients with stage IIIB or Stage IV NSCLC [5, 6]. The ASCO guidelines for the treatment of advanced NSCLC recommends platinum-based combination chemotherapy be administered for no more than six cycles as more cycles do not provide any survival benefit and have a higher risk of toxicity [7]. This recommendation was based on the results of randomized trials that compared shorter versus longer periods of administration of platinum-based chemotherapy [8–11]. New guidelines recommend maintenance therapy as a standard treatment for these patients post first-line therapy.

Randomized controlled trials of patients with stage IV disease and good PS have shown that platinum based doublet chemotherapy improves survival and palliates disease-related symptoms. Patients with non-squamous cell histology, good PS, no history of hemoptysis or other bleeding, or recent history of cardiovascular events may benefit from the addition of bevacizumab to paclitaxel and carboplatin. Patients with tumors harboring mutations in EGFR, particularly those from East Asia, never smokers, and those with adenocarcinoma may benefit from EGFR tyrosine kinase inhibitors as an alternative to first- or second-line chemotherapy. The use of thesecombinations of drugs is based on tumour histology and genetic mutations along with patient factors like age, co-morbidities and performance status. Molecular aberrations highly responsive to kinase inhibitors arise only in a minority ofpatient cases. For the majority of patients, cytotoxic chemotherapy isthe mainstay of treatment, with response rates to platinum-based combinations ranging from 20% to 35%. After four to six cycles of first-line or induction chemotherapy, approximately two thirds of patients have non-progressive disease. Continuation of first-line platinum-based combination regimens beyond four to six cycles in the past have resultedin heightened toxicities and diminished quality of life without providing a survival advantage. Thus, the standard therapeutic approach has entailed stopping treatment at that point, close clinical and radiographic surveillance, and initiation of second-line treatment at the time of progression.

For non-responding or progressing patients, second-line chemotherapy withdocetaxel, pemetrexed, erlotinib or gefitinib offers control of disease-related symptoms, improves quality of life, and prolongs overall survival (OS) [12-16]. However, only a select subgroup of patients (<50%) go on to receive second-line therapy due to side-effects of 1st line chemotherapy or low performance status. Therefore exploration of a maintenance strategy has been a sensible development.

Maintenance chemotherapy

The U.S. National Cancer Institute's medical dictionary defines maintenance therapy as "any treatment that is given to keep cancer from progressing after it has been successfully controlled by the appropriate front-line therapy; it may include treatment with drugs, vaccines or antibodies, and it should be given for a long time". In advanced NSCLC, it refers to the systemic therapy that may be given after 4-6 cycles of 1st line cytotoxic chemotherapy. Different approaches that might be classified as maintenance therapy in advanced NSCLC include: (1) continuing only the non-platinum or molecularly targeted component of the induction regimen, also known as 'continuation maintenance'; and (2) switching to a different cytotoxic or molecularly targeted agent, often called 'switch maintenance'. As per NCCN guidelines, maintenance therapy is astandard treatment option for select patients with partial or complete response to previous therapy or stable disease and is not considered standard of care for patients with ECOG PS 3-4 or with progressive disease [18].

Central to the evolution of the maintenance treatment strategy is the Goldie Coldman hypothesis [19], which dictates that even the smallest detectable cancers contain at least one drug-resistant clone and that increasing numbers of resistant clones emerge as tumours grow and progress. To overcome this phenomenon, the use of different non-cross-resistant chemotherapy regimens in alternating or sequential fashion has been employed [20, 21]. This samehypothesis underpins the design of maintenancetrials in which NSCLC patients are switched to a new, potentially non-cross-resistant agent if they respond to or remain stable on initial therapy. This 'switch maintenance' therapy is the most closely examined and has become the most debated [22]. Potential rationales for maintenance therapy include increased exposure to effective therapies, decreasing chemotherapy resistance,

TABLE 1: Clinical experience with maintenance therapy in advanced nsclc: summary of selected phase iii trials

Author/Year	Agent Vs Control	Patient no.	Progression free Survival	Salvage Rx %	Overall survival
Fidias *et al*. in 2009 [23]	Docetaxel 75 mg/m2 every 3 Weeks for up to 6 cyclesVs. Observation	153 Vs. 156	5.7 m Vs. 2.7 mHR: 0.63, P<.001	63	12.3 m Vs. 9.7 m HR: 0.80 , P =.085
JMEN (Ciuleanu *et al*.) in 2009 [24]	Pemetrexed 500 mg/ m2 every 3 weeks + BSC Vs. Placebo + BSC	441 Vs. 222	4.0 m Vs. 2.0m HR: 0.60 , P < .001	67	13.4 m Vs. 10.6 m , HR: 0.79 , P = .012
SATURN (Capuzzo *et al*.) in 2010 [25]	Erlotinib 150 mg PO daily Vs. Placebo	438 Vs. 451	12.3 wks Vs. 11.1 wks HR: 0.71 P < .001	72	12 m Vs. 11 m HR: 0.81 P = .0088
ATLAS (Miller *et al*.) in 2009 [26],[27]	Bevacizumab 15 mg/kg every 3 wks + erlotinib 150 mg PO daily Vs. Bevacizumab 15 mg/kg every 3 wks + placebo	373 Vs. 370	4.8 m Vs. 3.8 m HR: 0.72 P = .0012	55.5%	15.9 m Vs. 13.9 m , HR: 0.9 P = .2686
IFCT (Perol *et al*.) in 2010 [28]	Erlotinib 150 mg PO daily Vs. Observation	155 Vs. 155	2.9 m Vs. 1.9 m HR: 0.69 P = .003	81.9%	11.8 m Vs. 10.7 m , HR: 0.87 P = .3
INFORM (Zhang *et al*.) in 2011 [29]	Gefitinib 250 mg PO daily Vs. Placebo	148 Vs.148	4.8 m Vs. 2.6 m HR: 0.42 P < .0001	58.8	18.7 m Vs. 16.9 m , HR: 0.84 P = .2608

optimizing efficacy of chemotherapeutic agents, anti-angiogenic effects, and altering antitumor immunity. With switch maintenance, effective drugs are delivered to a substantially higher proportion of patients, generally more than 90% as compared to <50% when a second-line approach is used [21].

Maintenance therapy after 4-6 cycles of platinum doublet chemotherapy has shown an improvement in PFS and an improvement in OStoo.

The JMEN study compared maintenance chemotherapy with pemetrexed with placebo in stable and responding patients treated initially with one of three platinum-based regimens [24]. Stable and responding patients were randomized 2:1 to receive pemetrexed or placebo. There were 660 patients randomized and an analysis by histology was a pre-specified endpoint. Of the patients randomized to pemetrexed, 48% received 6 cycles and 23% - 10 cycles. There was a significant PFS advantage seen in the group as a whole(HR 0.6, p¼0.00001). Subgroup analysis revealed that patients with non-squamous histology had a HR of 0.47 (p¼0.00001, interaction *p*-value 0.036). When OS was examined, there remained a significant advantage in the entire treatment group (HR 0.79, p¼0.012). Furthermore, patients with non-squamous tumour had a median survival advantage of 5 months (15.5 versus 10.3 months) with a significant OS benefit (HR 0.7, p¼0.002, interaction *p*-value 0.033). This finding that pemetrexed improves survival in maintenance setting in non-squamous carcinoma was confirmed in the next study called PARAMOUNT study [32, 33]. Regulatory bodies have approved pemetrexed as maintenance chemotherapy in non-squamous NSCLC.

The strategy of maintenance therapy with targeted agents has been evaluated in a number of phase III trials. Three studies have used the EGFR inhibitor erlotinib [25, 26, 28] and three have used the EGFR inhibitor gefitinib [29–31]. The sequential Tarceva in unresectable non-small cell lung cancer trial (SATURN) was a large international study in which 1949 patientswere treated initially with four cycles of platinumbased chemotherapy [25]. Stable and responding patients (*n*-889) were randomized to receive maintenance erlotinib or placebo. The primary endpoint was PFS and patients were stratified by a number of clinical factors as well as by EGFR protein expression (assessed by immunohistochemistry [IHC]) and EGFR gene copy assessed by fluorescent in-situ hybridization (FISH). Both PFS and OS were significantly longer in the erlotinib arm (HR for PFS 0.71, *p*<0.0001; HR for OS

0.81, *p*-0.0088) resulting in its regulatory approval for this indication. Erlotinib approval for maintenance therapy has not been linked to any EGFR marker in either the United States or Europe.

In advanced stage NSCLC where systemic palliative therapy has limited survival benefit, to maintain or improve patients' quality of life (QOL) represents one of the main treatment goals. Several tools have been developed for measuring quality of life in cancer patients, such as the Functional Living Index-Cancer (FLIC) [34], Functional assessment of cancer therapy (FACT) scale [35], lung cancer symptom scale (LCSS) [36], and the European Organization for Research and Treatment of Cancer (EORTC) QLQ questionnaires which will be used in the present study [37]. The EORTC QLQ is a reliable, valid and clinically relevant tool for assessing lung cancer patients [38] and is efficacious for use in clinical trials as seen in comparative studies [39]. The EORTC QLQ-C30 includes five functional scales (physical, role, emotional, cognitive, and social), three symptom scales (fatigue, nausea or vomiting, and pain), global health status, and six single items (dyspnea, insomnia, appetite loss, constipation, diarrhea, and financial difficulties) [40]. The QLQ LC-13 is a supplementary, lung cancer specific questionnaire with 13 items addressing symptoms associated with lung cancer and its standard treatment [41].

Studies with QOL analysis in patients of advanced NSCLC as a primary outcome are limited and some have shown benefit in patients treated in first line setting [42]. Quality of life has also been studied in maintenance therapy. Belani et al. (2012) prospectively analyzed QOL of patients treated in JMEN study and found that patients receiving maintenancepemetrexed had significantly longer time toworsening of pain and hemoptysis symptoms[43]. The PARAMOUNT study also reported similar results [44]. Patients on the SATURN study showed significant delay in the time to pain and analgesic use in those receiving erlotinib maintenance[45]. Based on these multiple QOL analyses accompanying their respective trials, it appears that maintenance therapy in NSCLC delays the worsening of QOL and is beneficial in terms of QOL.

To summarize, there are two drugs approved in the maintenance setting in non-small cell lung cancer-pemetrexed and erlotinib. These two drugs have different side effect profile. Usually oral treatment is considered safer and more convenient than IV chemotherapy but this has not been compared in the maintenance setting. We want to compare the effect of these two drugs which are both standard of care, on the quality of life of the patients.

AIMS AND OBJECTIVES

Outcomes

Primary: Study the QOL in these two regimes at 3 months.

Secondary: Safety and Toxicity of these two regimens PFS and OS in these two regimens

MATERIALS AND METHODS

Study design

This is a prospective randomized open label study comparing Pemetrexed and Erlotinib in maintenance therapy of Non-squamous NSCLC. The protocol is required to be approved by Institutional Review Board at Tata Memorial Hospital.

Study population

Inclusion criteria shall be:

1. TKI-naive patients with documented Stage III OR Stage IV NSCLC who experience stable disease/ partial response/ complete response after 4-6 cycles of Pemetrexed + Cisplatin/Carboplatin chemotherapy.
2. ECOG Performance status (PS) from 0 to 2
3. Age ≥ 18yrs
4. Adequate bone marrow tests (absolute neutrophil count >1500/lL, hemoglobin > 8 g/dL, and platelet count >100,000/lL), renalfunction tests (creatinine<2 mg/dL), and liver functiontests (total bilirubin <1.5 times the institutional upperlimit of normal [ULN], aspartate aminotransferase and alanine aminotransferase levels <2 times the institutional ULN);
5. Life expectancy ≥ 3 months
6. Clinically stable brain metastases if any

Exclusion criteria shall be:

1. Prior therapy with TKI
2. Squamous cell Histology
3. A second primary tumor
4. Uncontrolled infection
5. Uncontrolled co-morbidities

Premenopausal women will be required to practice a suitable method of birth control and have a negative pregnancy test.

Treatment drugs

Pemetrexed: Dose - 500mg/m2 IV once in every 3 weeks

It is a pyrrolpyrimidineantifolate analog which acts mainly by inhibition of folate dependent enzyme thymidylatesynthetase (TS) resulting in inhibition of DNA synthesis and function. It also inhibits dihydrofolate reductase and two formyltransferases. It is metabolized intracellularly to more potent polyglutamates. It is principally excreted by kidneys. Its dose-limiting toxicity is myelosuppression. Other toxicities include skin rashes and hand-foot syndrome, nausea, vomiting, diarrhea,mucositis, fatigue, muscle or joint ache, motor or sensory neuropathy and transient derangement of liver function tests.

Erlotinib: Dose - 150mg PO once daily

It is a potent and selective EGFR tyrosine kinase inhibitor causing inhibition of critical mitogenic and anti-apoptotic signals involved in proliferation, growth,

metastasis, angiogenesis and response to chemotherapy and/or radiation therapy. Oral bioavailability is up to 100% after food. It is 90% bound to plasma proteins. Erlotinib is primarily metabolized in liver by the microsomal enzyme system and excreted in feces. Side effects of erlotinib treatment includes diarrhea, nausea, vomiting, oral ulcers, skin rashes with itching, acne and pustules, painful fissures or cracking of skin of hands and feet, alopecia and hair growth abnormalities, nail changes including paronychia and brittle or loose nails, fatigue, loss of appetite, conjunctivitis, abnormal LFTs, elevated INR and bleeding events, and rarely breathlessness, fever, cough and interstitial lung disease.

Concomitant drugs

Concomitant medications are allowed in the study patients as per their co-morbidities and symptomatic management. Vitamin B12 1000 mcg 1 week prior to beginning pemetrexed and every 9 weeks thereafter, daily folic acid supplementation and dexamethasone for 3 days starting 1 day before pemetrexed will be given in the pemetrexed arm. GCSF may be needed in pemetrexed arm. Zoledronic acid for bony metastatic lesions and palliative radiation for painful or symptomatic metastasis will be given.

Dose modification and treatment interruption

Pemetrexed needs to be withheld when creatinine clearance is reduced to less than 45ml/min from baseline. There is no recommended dose reduction for hepatic dysfunction. In case of Grade III/IV neutropenia or thrombocytopenia, dose reduction is required as per standard practice.

For Erlotinib, dose reduction is considered in patients with severe hepatic dysfunction and/or in those with a bilirubin > 3 times the upper limit for normal or AST/ALT > 5 times the upper limit for normal. No dose reduction is required for renal dysfunction. Erlotinib dose reduction or temporary interruption may be needed in Grade III/IV diarrhea and skin reactions. It should be withheld if ILD is suspected and discontinued if proven.

Discontinuation of treatment

Treatment will be discontinued any time when progression is detected , quality of life or performance scale deteriorates during therapy, serious or intolerable side-effects occur as a result of treatment or if the participant wishes as such due to any reason.

Tumour assessment

Tumour response assessment post 1st line chemotherapy before starting maintenance therapy and during the maintenance phase as per routine practice would be done radiologically with scan as when needed by the treating physician.

Efficacy, safety and toxicity assessment

Patients will be followed up in OPD as per standard care. QOL (Quality of Life) assessment will be done at baseline and at 3 months using the EORTC-QLQ-C30 and LC13 questionnaire for lung cancer. Any adverse event whether related or unrelated to treatment would be recorded in the Adverse events form with grade as per CTCAE 4.3. Number of days of hospitalization required during the therapy will also be noted.

STATISTICAL ANALYSIS

Randomization

The patients shall be stratified into Pemetrexed and Erlotinib treatment arms by block randomization so that the two groups are comparable.

Sample size

Primary outcome is change in the score of QOL at 3 months We estimate that with 200 patients, the study will have 80% power to detect a significant difference between the two groups with an alpha error of 5%, when the effect is 0.3 [46].

STATISTICAL METHODS

Changes in HRQOL scores during the study will be calculated as the difference between baseline and 3 months' value and compared using the paired *t* test. Effect sizes will be calculated by dividing the changes in each HRQOL score by the standard deviation (SD) of that score estimated at baseline on the entire sample. Analysis of the study variables will be done using simple percentages, log rank test, cox proportional regression analysis and Kaplan Meir Curves.

PRIMARY END POINT: 3 months

Quality of life of patients in the two arms of the study shall be determined using the EORTC-QLQ-C30 and LC13 questionnaire for lung cancer. QOL assessment shall be done at every OPD visit.

SECONDARY END POINTS: Discontinuation of treatment or death due to any cause

Any serious adverse event occurring after the patient has provided informed consent and until four weeks after the patient has stopped study participation shall be reported to the ethics committee. Information about all serious adverse events will be collected and recorded on the Adverse Events Report Form. All the adverse events shall be graded as per the CTCAE Version 4.03. Adverse events recorded in the two study arms shall be compared.

Overall survival in these two arms shall be compared during the period of study. Overall survival will be calculated from the date of diagnosis to date of death from any cause.

Feasibility of the study

We register approximately 1300 patients of lung cancer at TMH. Approximately 1000 patients is planned for palliative systemic therapy. 700 patients are non-squamous NSCLC. We expect 25% of the patients to enroll in the study. We expect to complete the study in 2 years.

Ethical considerations

Both the study arms are standard of care at TMH. Both the drugs are approved for NSCLC. Patients in both arms will receive standard of care.

Informed consent

Each participant shall be informed of the objectives, benefits, risks and requirements of the study. The participants shall be provided with an information sheet in clear, simple language that they understand. The informed consent will be obtained from the patient after the patient clearly reads and understands the informed consent and the patient's doubts and queries are answered. Since patient will be receiving standard of care in both arms, if complications arise from the trial, they will be managed routinely and the patient will bear the cost. All patient information collected will be confidential and will be available only to the investigators and treating physicians.

1. SCHEME OF STUDY

Advanced NSCLC (non-squamous) Post 4-6 cycles of Pemetrexed + Platinum with stable disease or response to therapy and willing for switch maintenance therapy

↓

Fit for study + willing for study

↓

Informed Consent

↓

Randomization into two treatment arms

↓

Continuation of treatment till decline in QOL or Poor PS or disease progression or significant toxicity or death

↓

Primary: Quality of Life in 2 arms at 3 months

Secondary: Adverse Events in 2 arms, OS in 2 arms

REFERENCES

1. Ferlay J, Soerjomataram I, Ervik M, Dikshit R, Eser S, Mathers C, Rebelo M, Parkin DM, Forman D, Bray, F. GLOBOCAN 2012 v1.0, Cancer Incidence and Mortality Worldwide: IARC CancerBase No.11. Lyon, France: International Agency for Research on Cancer. 2013. Available from http://globocan.iarc.fr. https://doi.org/10.1002/ijc.29210.
2. Noronha V, Dikshit R, Raut N, Joshi A, Pramesh C S, George K, Agarwal J P, Munshi A, Prabhash K. Epidemiology of lung cancer in India: Focus on the differences between non-smokers and smokers: A single-centre experience. Indian J Cancer 2012;49:74-81
3. Felip E, Stahel RA, Pavlidis N; ESMO Guidelines Task Force. ESMOMinimum Clinical Recommendations for diagnosis, treatment andfollow-up of non-small-cell lung cancer (NSCLC). Ann Oncol. 2005;16: i28–29. https://doi.org/10.1093/annonc/mdi821. [PubMed]
4. Fleming ID, Cooper JS, Henson DE. Lung. In: Fleming ID,Cooper JS, Henson DE, et al, eds. AJCC cancer staging manual.5th edn. Philadelphia: Lippincott-Raven, 1997: 127–37.
5. Chemotherapy in non-small cell lung cancer-a meta-analysis using updated data on individual patients from 52 randomized clinical trials. Non-Small Cell Lung Cancer Collaborative Group. BMJ. 1995; 311:899–909. https://doi.org/10.1136/bmj.311.7010.899.
6. Grilli R, Oxyman A, Julian J. Chemotherapy for advanced non-smallcell lung cancer: how much benefit is enough? J ClinOncol. 1993; 11:1866–1872. https://doi.org/10.1200/JCO.1993.11.10.1866. [PubMed]
7. Azzoli, C. G., Baker Jr, S., Temin, S., Pao,W., Aliff, T., Brahmer, J. et al. (2009) American Society of ClinicalOncology Clinical Practice Guideline update on chemotherapyfor stage IV non-small-cell lung cancer. J ClinOncol 27: 6251-6266. https://doi.org/10.1200/JCO.2009.23.5622. [PubMed]
8. Park JO, Kim SW, Ahn JS, Suh C, Lee JS, Jang JS, Cho EK, Yang SH, Choi JH, Heo DS, Park SY, Shin SW, Ahn MJ, et al. Phase III trial of two versus four additional cycles in patients who are nonprogressive after two cycles of platinum-based chemotherapy in non small-cell lung cancer. J Clin Oncol. 2007; 25:5233–9. https://doi.org/10.1200/JCO.2007.10.8134. [PubMed]
9. von Plessen C1, Bergman B, Andresen O, Bremnes RM, Sundstrom S, Gilleryd M, Stephens R, Vilsvik J, Aasebo U, Sorenson S. Palliative chemotherapy beyond three courses conveys no survival or consistent quality-of-life benefits in advanced non-small-cell lung cancer. Br J Cancer. 2006; 95:966–73. https://doi.org/10.1038/sj.bjc.6603383. [PubMed]
10. Smith IE1, O'Brien ME, Talbot DC, Nicolson MC, Mansi JL, Hickish TF, Norton A, Ashley S. Duration of chemotherapy in advanced non-small-cell lung cancer: a randomized trial of three versus six courses of mitomycin, vinblastine, and cisplatin. J Clin Oncol. 2001; 19:1336–43. https://doi.org/10.1200/JCO.2001.19.5.1336. [PubMed]
11. Socinski MA, Schell MJ, Peterman A, Bakri K, Yates S, Gitten R, Unger P, Lee J, Lee JH, Tynan M, Moore M, Kies MS. Phase III trial comparing a defined duration of therapy versus continuous therapy followed by second-line therapy in advanced-stage IIIB/IV non-small-cell lung cancer. J Clin Oncol. 2002; 20:1335–43. https://doi.org/10.1200/JCO.2002.20.5.1335. [PubMed]
12. Shepherd FA, Dancey J, Ramlau R, Mattson K, Gralla R, O'Rourke M, Levitan N, Gressot L, Vincent M, Burkes R, Coughlin S, Kim Y, Berille J. Prospective randomized trial ofdocetaxel versus best supportive care in patients with non-small-cell lungcancer previously treated with platinum-based chemotherapy. J Clin Oncol. 2000; 18:2095–103. https://doi.org/10.1200/JCO.2000.18.10.2095. [PubMed]
13. Fossella FV, DeVore R, Kerr RN, Crawford J, Natale RR, Dunphy F, Kalman L, Miller V, Lee JS, Moore M, Gandara D, Karp D, Vokes E, et al. Randomized phase III trial of docetaxelversus vinorelbine or ifosfamide in patients with advanced non-small-celllung cancer previously treated with platinum-containing chemotherapyregimens: the TAX 320 Non-Small Cell Lung Cancer Study Group. J Clin Oncol. 2000; 18:2354–62. https://doi.org/10.1200/JCO.2000.18.12.2354. [PubMed]
14. Hanna N, Shepherd FA, Fossella FV, Pereira JR, De Marinis F, von Pawel J, Gatzemeier U, Tsao TC, Pless M, Muller T, Lim HL, Desch C, Szondy K, et al. Randomized phase III trial ofpemetrexed versus docetaxel in patients with non-small-cell lung cancerpreviously treated with chemotherapy. J Clin Oncol. 2004; 22:1589–97. https://doi.org/10.1200/JCO.2004.08.163. [PubMed]
15. Shepherd FA, Rodrigues Pereira J, Ciuleanu T, Tan EH, Hirsh V, Thongprasert S, Campos D, Maoleekoonpiroj S, Smylie M, Martins R, van Kooten M, Dediu M, Findlay B, et al. Erlotinib in previouslytreated non-small-cell lung cancer. N Engl J Med. 2005; 353:123–32. https://doi.org/10.1056/NEJMoa050753. [PubMed]
16. Kim ES, Hirsh V, Mok T, Socinski MA, Gervais R, Wu YL, Li LY, Watkins CL, Sellers MV, Lowe ES, Sun Y, Liao ML, Osterlind K et al. Gefitinib versus docetaxel in previously treatednon-small-cell lung cancer (INTEREST): a randomised phase III trial. Lancet. 2008; 372:1809–18. https://doi.org/10.1016/S0140-6736(08)61758-4. [PubMed]
17. Hensing TA, Schell MJ, Lee JH, Socinski MA. Factors associatedwith the likelihood of receiving second line therapy for advancednon-small cell lung cancer. Lung Cancer 2005; 47:253–59. https://doi.org/10.1016/j.lungcan.2004.07.040. [PubMed]
18. NCCN Guidelines Version 2.2014 Non Small Cell Lung Cancer.
19. Goldie JH, Coldman AJ. A mathematicmodel for relating the drug sensitivity of tumors totheir spontaneous mutation rate. Cancer Treat Rep. 1979; 63:1727–1733. [PubMed]
20. Coate LE, Shepherd FA. Maintenance therapy in advanced non-small cell lung cancer: evolution, tolerability and outcomes. Ther Adv Med Oncol. 2011; 3:139–157. https://doi.org/10.1177/1758834011399306. [PubMed]
21. Gerber DE, Schiller JH. Maintenance chemotherapy foradvanced non-small-cell lung cancer: new life for an oldidea. J ClinOncol 2013; 31:1009–20. https://doi.org/10.1200/JCO.2012.43.7459. [PubMed]

22. Stinchcombe T. The role of maintenancetherapy in advanced non-small cell lung cancer. ASCOEducational Book. 2010.

23. Fidias PM, Dakhil SR, Lyss AP, Loesch DM, Waterhouse DM, Bromund JL, Chen R, Hristova-Kazmierski M, Treat J, Obasaju CK, Marciniak M, Gill J, Schiller JH. Phase III study of immediate compared with delayed docetaxel after front-line therapy with gemcitabine plus carboplatin in advanced non-small-cell lung cancer. J Clin Oncol. 2009; 27:591–8. https://doi.org/10.1200/JCO.2008.17.1405. [PubMed]

24. Ciuleanu T1, Brodowicz T, Zielinski C, Kim JH, Krzakowski M, Laack E, Wu YL, Bover I, Begbie S, Tzekova V, Cucevic B, Pereira JR, Yang SH, et al. Maintenance pemetrexed plus best supportive careversus placebo plus best supportive care for nonsmall-cell lung cancer: a randomised, double-blind,phase 3 study. Lancet. 374:1432–1440. https://doi.org/10.1016/S0140-6736(09)61497-5.

25. Cappuzzo F1, Ciuleanu T, Stelmakh L, Cicenas S, Szczésna A, Juhász E, Esteban E, Molinier O, Brugger W, Melezínek I, Klingelschmitt G, Klughammer B, Giaccone G; SATURN investigators. Erlotinib as maintenance treatment in advanced non-small-cell lung cancer: a multicentre, randomised, placebo-controlled phase 3 study. Lancet Oncol. 2010; 11:521–9. https://doi.org/10.1016/S1470-2045(10)70112-1.

26. Miller VA, O›Connor P, Soh C, et al: A randomized,double-blind, placebo-controlled, phase IIIbtrial(ATLAS) comparing bevacizumab (B) therapy with orwithout erlotinib (E) after completion of chemotherapywith B for first-line treatment of locally advanced,recurrent, or metastatic non-small cell lungcancer (NSCLC). J ClinOncol. 2009; 27:407s. https://doi.org/10.1200/jco.2009.27.18s.lba8002.

27. Kabbinavar FF, Miller VA, Johnson BE, O'Connor PG, Soh C. ATLAS Investigators. Overall survival (OS) in ATLAS, a phase IIIb trialcomparing bevacizumab (B) therapy with or withouterlotinib (E) after completion of chemotherapy(chemo) with B for first-line treatment of locally advanced, recurrent, or metastatic non-small celllung cancer (NSCLC). J Clin Oncol. 2010; 28:544s. https://doi.org/10.1200/jco.2010.28.15_suppl.7526.

28. Pérol M1, Chouaid C, Pérol D, Barlési F, Gervais R, Westeel V, Crequit J, Léna H, Vergnenègre A, Zalcman G, Monnet I, Le Caer H, Fournel P, et al. Randomized, phase III study of gemcitabine or erlotinib maintenance therapy versus observation, with predefined second-line treatment, after cisplatingemcitabineinduction chemotherapy in advancednon-small-cell lung cancer. J Clin Oncol. 2012; 30:3516–3524.

29. Zhang L1, Ma S, Song X, Han B, Cheng Y, Huang C, Yang S, Liu X, Liu Y, Lu S, Wang J, Zhang S, Zhou C. Gefitinib versus placebo as maintenance therapy in patients with locally advanced or metastatic non-small-cell lung cancer (INFORM; C-TONG 0804): a multicentre, double-blind randomised phase 3 trial. Lancet Oncol. 2012; 13:466–465. https://doi.org/10.1016/S1470-2045(12)70117-1. [PubMed]

30. Takeda K1, Hida T, Sato T, Ando M, Seto T, Satouchi M, Ichinose Y, Katakami N, Yamamoto N, Kudoh S, Sasaki J, Matsui K, Takayama K, et al. Randomized phase III trial of platinum-doublet chemotherapy followed by gefitinib compared with continued platinum-doublet chemotherapy in Japanese patients with advanced non-small-cell lung cancer: results of a west Japan thoracic oncology group trial (WJTOG0203). J Clin Oncol. 2010; 28:753–760. https://doi.org/10.1200/JCO.2009.23.3445. [PubMed]

31. Gaafar RM, Surmont VF, Scagliotti GV, Van Klaveren RJ, Papamichael D, Welch JJ, Hasan B, Torri V, van Meerbeeck JP; EORTC Lung Cancer Group and the Italian Lung Cancer Project. A double-blind, randomised, placebo-controlled phase III intergroup study of gefitinib in patients with advanced NSCLC, non-progressing after first line platinum-based chemotherapy (EORTC 08021/ILCP 01/03). Eur J Cancer. 2011; 47:2331–2340. https://doi.org/10.1016/j.ejca.2011.06.045. [PubMed]

32. Paz-Ares L1, de Marinis F, Dediu M, Thomas M, Pujol JL, Bidoli P, Molinier O, Sahoo TP, Laack E, Reck M, Corral J, Melemed S, John W, et al. Maintenance therapy with pemetrexed plus best supportive care versus placebo plus best supportive care after induction therapy with pemetrexed plus cisplatin for advanced non-squamous non-small-cell lung cancer (PARAMOUNT): a double-blind, phase 3, randomised controlled trial. Lancet Oncol. 2012; 13:247–255. https://doi.org/10.1016/S1470-2045(12)70063-3. [PubMed]

33. Paz-Ares L, De Marinis F, Dediu M, et al: PARAMOUNT: Final overall survival (OS) results ofthe phase III study of maintenance pemetrexed(pem) plus best supportive care (BSC) versus placebo (plb) plus BSC immediately following inductiontreatment with pem plus cisplatin (cis) for advancednonsquamous (NS) non-small cell lung cancer (NSCLC). J Clin Oncol. 2012; 30:481s. https://doi.org/10.1200/jco.2012.30.18_suppl.lba7507.

34. Morrow GR1, Lindke J, Black P. Measurement of quality of life in patients: psychometric analyses of the functional living index-cancer (FLIC). Quality of Life Research. 1992; 1:287–296. https://doi.org/10.1007/BF00434942. [PubMed]

35. Cella DF1, Tulsky DS, Gray G, Sarafian B, Linn E, Bonomi A, Silberman M, Yellen SB, Winicour P, Brannon J. The functional assessment of cancer therapy (FACT) scale: Development and validation of the general measure. J Clin Oncol. 1993; 11:570–9. https://doi.org/10.1200/JCO.1993.11.3.570. [PubMed]

36. Hollen PJ1, Gralla RJ, Kris MG, Cox C, Belani CP, Grunberg SM, Crawford J, Neidhart JA. Measurement of quality of life in patients with lung cancer in multicenter trials of new therapies. Psychometric assessment of the Lung Cancer Symptom Scale Cancer. (1994; 73:2087–2098. https://doi.org/10.1002/1097-0142(19940415)73:8<2087::AID-CNCR2820730813>3.0.CO;2-X.

37. Aaronson NK, Ahmedzai S, Bergman B, Bullinger M, Cull A, Duez NJ, Filiberti A, Flechtner H, Fleishman SB, de Haes JC. The European Organization for Research and Treatment of Cancer QLQ-C30: a quality-of-life instrument for use in international clinical trials in oncology. J Natl Cancer Inst. 1993; 85:365–76. https://doi.org/10.1093/jnci/85.5.365. [PubMed]

38. Nicklasson M, Bergman B. Validity, reliability and clinical relevance of EORTCQLQ-C30 and LC13 in patients with chest malignancies in a palliative setting. Qual Life Res. 2007; 16:1019–28. https://doi.org/10.1007/s11136-007-9210-8. [PubMed]

39. Luckett T, King MT, Butow PN, Oguchi M, Rankin N, Price MA, Hackl NA, Heading G. Choosing between the EORTC QLQ-C30 and FACT-G formeasuring health-related quality of life in cancer clinicalresearch: issues, evidence and recommendations. Ann Oncol. 2011; 22:2179–90. https://doi.org/10.1093/annonc/mdq721. [PubMed]

40. Aaronson NK, Ahmedzai S, Bergman B, Bullinger M, Cull A, Duez NJ, Filiberti A, Flechtner H, Fleishman SB, de Haes JC. The European organization for research and treatment of cancer QLQ-C30: A quality-of-life instrument for use in international clinical trials in oncology. J Natl Cancer Inst. 1993; 85:365–76. https://doi.org/10.1093/jnci/85.5.365. [PubMed]

41. Bergman B, Aaronson NK, Ahmedzai S, Kaasa S, Sullivan M. The EORTC QLQ-LC13: a modular supplement to the EORTC Core Quality of Life Questionnaire (QLQ-C30) for use in lung cancer clinical trials. EORTC Study Group on Quality of Life. Eur J Cancer. 1994; 30A:635–42. https://doi.org/10.1016/0959-8049(94)90535-5. [PubMed]

42. Chu D, Nguyen J, Koo K, Zeng L, Bedard G, Lam H, Wong E, Popovic M, Chow E. An Update on the Quality of Life Measurements in Lung Cancer Patients Receiving Palliative Radiotherapy: A Literature Review. World J Oncol. 2013; 4:67–73.

43. Belani CP1, Brodowicz T, Ciuleanu TE, Krzakowski M, Yang SH, Franke F, Cucevic B, Madhavan J, Santoro A, Ramlau R, Liepa AM, Visseren-Grul C, Peterson P, et al. Quality oflife in patients with advanced non-small-cell lungcancer given maintenance treatment with pemetrexedversus placebo (H3E-MC-JMEN): results from arandomised, double-blind, phase 3 study. Lancet Oncol. 2012; 13:292–9. https://doi.org/10.1016/S1470-2045(11)70339-4.

44. Gridelli C, de Marinis F, Pujol JL, Reck M, Ramlau R, Parente B, Pieters T, Middleton G, Corral J, Winfree K, Melemed S, Zimmermann A, John W, et al. Safety, resource use, and quality of life in paramount: a phase III study of maintenance pemetrexed versus placebo after induction pemetrexed plus cisplatin for advanced nonsquamous non-small-cell lung cancer. J Thorac Oncol. 2012; 7:1713–1721. https://doi.org/10.1097/JTO.0b013e318267cf84. [PubMed]

45. Juhász E1, Kim JH, Klingelschmitt G, Walzer S. Effects of erlotinib first-line maintenancetherapy versus placebo on the health-related qualityof life of patients with metastatic non-small-cell lungcancer. Eur J Cancer. 2013; 49:1205–15. https://doi.org/10.1016/j.ejca.2012.11.006. [PubMed]

46. Temel JS1, Greer JA, Muzikansky A, Gallagher ER, Admane S, Jackson VA, Dahlin CM, Blinderman CD, Jacobsen J, Pirl WF, Billings JA, Lynch TJ. Early palliative care for patients with metastatic non-small-cell lung cancer. N Engl J Med. 2010; 363:733–742. https://doi.org/10.1056/NEJMoa1000678. [PubMed]
